# Compulsion for Medical Men?

**Published:** 1917-01-13

**Authors:** 


### COMPULSION FOR MEDICAL MEN?

How to supply the need of more military medical officers is a question that has come to the fore of late; and from both inside and outside the profession the suggestion of a sort of conscription has been made. This special conscription would be at least as complicated a matter, as full of reverberations, as the measure has been found to be in general. It is safe to assume that although, for a country engaged in a great war, the health and recuperation of its army are more important than those of its non-combatant population, yet this primary consideration must be offset by the economic necessity of not letting the physical efficiency of civil workers suffer, and by the strong claims to hygienic care of the rising generation. What data exist by which to arrive at practical decisions, from which to calculate how many doctors exactly shall be sent to the field and how many kept at home? The answer must be—none; because in England such an emergency has never arisen since medicine escaped from mediævalism. Therefore, before saying what ought or ought not to be done now, it were best to get some of the facts of the situation tabled as soon as possible. Readers may care to have particulars of a candid and detailed report which has reached us of what has actually happened up to date as regards the point in question in a certain urban district.

It is a good one for the present purpose, being typical of many: an industrial town of fifty-odd thousand inhabitants such as is found all over the Midlands, the North of England, and the South of Wales and Scotland. Here, before the war broke out, were found some twenty-five doctors. Twelve of these went sooner or later to the war, three of them for a limited period; but one of the three is now liberating his partner for military service. Of the thirteen who remained, three are over age, and one not of unmixed European parentage. Another has a heavy rent to pay and cannot afford the pecuniary sacrifice entailed in taking a commission; a sixth is unable to leave home on account of domestic affairs that urgently call for his presence there; while a seventh has a partner who is enough of an invalid to undertake no night work. There is, further, the Medical Officer of Health, who has been refused permission to leave by his committee. Thus five are left; but if these five were sent off *nolens volens* the eight previously specified could not do nearly all the town's medical work, which comprises, incidentally, that of about 150 hospital beds. As regards emoluments due to absentees, things are going fairly harmoniously, the arrangement being that the practitioner who is away receives half the fees of his practice. In one instance, that of two neighbours whose practices cover one quarter of the town, a mutual agreement was reached whereby both served, and so neither benefited by the other's absence. In another similar one, the younger man left it to his senior to go. In equity, compulsion should be applied here and the senior brought back; but obviously no military advantage would result. Nor, perhaps, would the one or two medical men that the sick population of this town could spare repay the disturbance and extra organising work that the institution of medical conscription would mean.

Of course, this is but one sample of a very big batch. But everywhere there are many constant factors. Human nature is practically constant. It is, too, a single country we are considering, and a single profession, and a single stimulus. It is not likely that the reaction has been much less uniform. These general practitioners—all of them, save one or two, on the panel—have probably behaved in sum very much as an equal number of general medical practitioners have behaved everywhere else in industrial districts, where three-quarters of the national population are concentrated. Probably, therefore, from the ranks of general practice very few more army doctors can be drawn. General practitioners form the vast majority of the medical profession. They, and operating surgeons, fulfil the medical work which is absolutely necessary to the community. The same cannot be said of the medical consultant, especially in the provinces, for even before the war surgical clinicians were making out a very good case for being allowed more hospital beds than their medical colleagues, whose work not only they, but also bacteriologists and clinical specialists, had encroached upon so much. Of course, nineteen-twentieths of war work at home is surgical, at least does not appertain to the general physician. For the short period probably remaining before hostilities are suspended, the community would not suffer if provincial medical education were suspended, and students and their medical teachers set free to act as dressers and anæsthetists abroad.

